# Motives and barriers to safer sex and regular STI testing among MSM soon after HIV diagnosis

**DOI:** 10.1186/s12879-017-2277-0

**Published:** 2017-03-07

**Authors:** Titia Heijman, Freke Zuure, Ineke Stolte, Udi Davidovich

**Affiliations:** 1 STI outpatient Clinic, Amsterdam Public Health Service, Amsterdam, The Netherlands; 2 Department Infectious Diseases, Research and Prevention, Amsterdam Public Health Service, Amsterdam, The Netherlands; 3 Centre for Infection and Immunology Amsterdam (CINIMA), Academic Medical Centre (University of Amsterdam), Amsterdam, The Netherlands; 4 University of applied sciences InHolland, Haarlem, The Netherlands

**Keywords:** Men who have sex with men, Recent diagnosis, HIV-positive, Condom use, STI testing

## Abstract

**Background:**

Understanding why some recently with HIV diagnosed men who have sex with men (MSM) choose for safer sex and regular STI testing, whereas others do not, is important for the development of interventions that aim to improve the sexual health of those newly infected.

**Methods:**

To gain insight into motives and barriers to condom use and regular STI testing among MSM soon after HIV diagnosis, 30 HIV-positive MSM participated in semi-structured qualitative interviews on sexual health behaviours in the first year after HIV diagnosis.

**Results:**

Typical barriers to condom use soon after diagnosis were emotions such as anger, relief, and feelings of vulnerability. Additional barriers were related to pre-diagnosis patterns of sexual-social behaviour that were difficult to change, communication difficulties, and substance use. Barriers to STI testing revolved around perceptions of low STI risk, faulty beliefs, and burdensome testing procedures.

**Conclusions:**

The great diversity of motives and barriers to condom use and STI testing creates a challenge to accommodate newly infected men with information, motivation, and communication skills to match their personal needs. An adaptive, tailored intervention can be a promising tool of support.

## Background

In 2014, 898 persons were newly diagnosed with HIV in the Netherlands, and men having sex with men (MSM) accounted for 68% of these diagnoses [1]. At the point of learning they are HIV-positive, MSM tend to reduce their sexual risk behaviour, but this reduction is only temporary and is shorter in the era of combination anti-retroviral therapy (cART) compared to the pre-cART era [2–5]. Even now that cART is available, men having condomless sex shortly after diagnosis may still have detectable virus loads, as treatment has just begun or has yet to be started. Thus their HIV-negative partners are at risk for HIV infection, and the men themselves are at risk for other sexually transmitted infections (STIs) [6–17]. In 2014, STIs were diagnosed in 34% of HIV-positive MSM who consulted the Amsterdam STI outpatient clinic, as opposed to 19% among its HIV-negative MSM clients [13]. Along with an undetectable viral load, condom use, and regular STI testing are beneficial for the health of HIV-positive MSM in preventing STI co-infections.

The aim of this study is to investigate the motives and barriers to safer sex, and the motives and barriers to regular STI testing, among MSM who have been recently diagnosed with HIV. Understanding why recently diagnosed men choose for condom use and regular STI testing, whereas others do not, will assist us in developing tailored interventions that are dedicated to support sexual health soon after diagnosis. Addressing the barriers to safe sex and STI screening as soon as possible after HIV diagnosis, and addressing the specific needs that are unique to that phase in time, should set the foundation for a healthier future sexual career. Such a tailored approach for addressing barriers for health behaviours has proven effective in the past [18, 19] including in the settings of HIV [20].

## Methods

### Recruitment and sample

From May 2008 to April 2009, MSM were recruited at the STI outpatient clinic of the Public Health Service of Amsterdam (PHSA), a large clinic performing annually about 9000 consultations for MSM. About 25% of its consultations are for HIV-positive men, and about 100 MSM are newly diagnosed with HIV each year [13]. We also recruited men from the Amsterdam Cohort Studies (ACS) among MSM, a longitudinal HIV seroconversion study. Its participants visit the PHSA every 6 months to provide detailed sexual behavioural information and blood samples for HIV testing [21].

In order to answer the research questions we chose to conduct interviews with two groups of men. The first group consisted of those who were diagnosed with HIV within 12 months prior to the interview; the second group consisted of men who were diagnosed longer than 12 months prior to the interview. The first group could provide present-life experiences, with less recall bias but with limited long-term perspective and experiences. On the other hand, men in the second group were interviewed retrospectively about their first year post-diagnosis and could therefore provide a more crystallized longer view of their past experiences. By recruiting men of different periods post diagnosis (<1 year<) we expected to enhance the in-depth rapport and understanding of possible perceptions and experiences behind perceived barriers and motives.

For those recently diagnosed with HIV, participation began at least 1 month after diagnosis, allowing them time to reflect on joining the study and increasing their possibility of having post-diagnosis sexual contact. For men diagnosed more than 12 months ago, we selected only those diagnosed after 1998, when cART had been widely available in the Netherlands for 2 years. We assumed this period provided sufficient time for reactive social and individual change to occur as a result of the introduction of cART.

During a consecutive period of 11 months, visitors of the STI clinic who were diagnosed at that visit for HIV and met the inclusion criteria were asked to participate in the study. These men received an information leaflet outlining the study purpose and the interview methods from an STI nurse. Men who were interested were asked to provide their contact details. After a month, a study researcher contacted the men, provided more detailed information, and made an appointment to interview men willing to participate. For anonymity, the interviews were not linked to clinical data of the PHSA. Recruitment continued until data saturation was reached and no new topics emerged from the interviews. The process resulted in a total of 30 interviews, 15 among recently diagnosed MSM and 15 among MSM more than 1 year post-diagnosis. The men received a 20-euro gift certificate as a reimbursement for participating.

### Interview procedures

A descriptive qualitative design was used to explore participant views and motives and barriers with regard to condom use and STI testing. Semi-structured interviews were chosen as they allow flexibility to explore new topics, facilitate empathy and trust, and can produce richer data [22]. Participants could choose between two interview methods: a classic face-to-face interview conducted at the PHSA or an online chat interview. The latter option was offered to reduce potential barriers to participation (e.g. traveling to the PHSA) and to enhance anonymity.

Interviews were conducted by researchers and nurses who were experienced with interviewing on sexual themes. There were 26 face-to-face interviews, each taking 60–90 min, and four online chat interviews, each taking 60–180 min. The interviewers were guided by a standardized interview manual which included the structural questions and the main topics as well as suggestions for open investigative questions.

### Theoretical background

The interviews themselves were guided by our goal to generate as much as phenomenological richness and experiences as possible, therefore we did not limit our interview schedule to a specific psycho-social theory. However, when interpreting our results we used two theoretical models to formulate and justify future recommendations how to address part of the barriers we describe. We interpreted our results for the discussion through two theories: the Information Motivation Behaviour skills model (IMB) [23] and the conceptual model for integrating social context in health-behaviour interventions [24]. In the discussion section we conceptualized our findings using relevant constructs of these theories in order to formulate specific recommendations for future HIV prevention and to maximize the chances that our suggestions will have beneficial impact on future behaviour.

According to the IMB model, information, motivation, and behavioural skills are interrelated determinants of behaviour. Applied to the context of sexual risk behavior and STI testing this model would suggest that the likelihood of initiate and maintain healthier behaviours is greatest when individuals are well informed, highly motivated, well skilled or have a positive sense of self-efficacy regarding the performance of safe sex behaviours and STI testing. The conceptual model for integrating social context in health-behaviour interventions focusses on social-context barriers (i.e. interpersonal, organizational, community, and societal). This model essentially suggests that safer sex and STI testing would increase when men experience societal and organizational facilitation towards these target behaviours.

### Interview schedule

Each interview started with a short explanation of the study. This was followed by a short list of questions regarding age, ethnicity, sexual orientation, cART use, STI diagnoses, and education. Ethnicity was operationalized as Dutch or non Dutch, sexual orientation as homosexual, bisexual or heterosexual, education as low, middle and high according to standard Dutch classification.

From there, a variety of topics was addressed to explore the men’s thoughts and emotions following their HIV diagnosis. They were asked to reflect in detail on their first sexual activity after diagnosis and their first incident of sexual risk (within 1 year of diagnosis). The motives and barriers to condom use were explored by asking for reasons why they did or did not use condoms or seek regular STI testing.

Follow-up questions were used to collect information on context and rationale. All face-to-face interviews were audio-taped and transcribed verbatim, and all online interviews were saved. Interviews were in Dutch. All quotes used in this paper were translated into English and a selection was made to represent the barrier or motive identified based on relevance and conciseness.

### Analyses

For the analyses of the transcripts we used Qualitative Data Analysis Software (MaxQDA2007). Analysis involved a team of three researchers with different social study backgrounds (anthropology, social and clinical psychology, and health communication).

After conducting the first two interviews, two researchers inductively coded the interviews, independently from one another. The process of open coding in which an unrestricted number of facets were expressed in preliminary code names was used. A discussion meeting involving the data analysis team took place to ensure that all relevant content was incorporated in codes.

The iterative process of interviewing alternated with open coding was repeated two times. In the second phase of the analysis, the researchers reduced and renamed the codes by focussing on the research questions. All 30 transcripts were then coded by the principal researcher, and grouped the relevant codes into categories.

### Ethical framework

The study was reviewed by the secretary of the Medical Ethical Committee of the Academic Medical Centre of Amsterdam, the Netherlands and received an exemption. In all interviews, transcriptions were anonymized prior to analysis by removing potential identifiers of the participants and their partners (i.e. first names). Interviews could not be linked to the electronic patient file of the STI outpatient clinic. Informed consent to record the interview was obtained orally (face-to-face-interview) or in writing (online chat-interview) before the interview started. Participants could withdraw from the study at any time without explanation.

## Results

### Descriptive

Of the 50 men who were asked to participate, 32 agreed to be contacted for the study; one participant withdrew and one could not be reached, resulting in 30 interviews. Table 1 shows the characteristics of the participants. Most participants (28/30) were born in the Netherlands, and all spoke Dutch. Median age at time of the interview was 38 years, varying from 25 to 54 years. Educational level was mainly high (high school or university). Half of the participants (*n* = 15) were recently diagnosed (<12 months) and were interviewed at a median time of 0.8 years (IQR 0.5–0.9 years) after HIV diagnosis. Those not recently diagnosed (*n* = 15) were interviewed at a median of 3.7 years (IQR 1.9–4.7 years) after diagnosis.

**Table 1:** Characteristics of the participants (*N* = 30)

	Total n/N	Percent
N participants	30	100
Sexual orientation		
Homosexual	30/30	100
Dutch nationality	28/30	93
Median age at time of HIV diagnose	38 (IQR 25–54)	
Level of education		
High	19/28^a^	68
Middle	6/28	21
Low	2/28	7
cART at time of interview	6/30	20
STI diagnoses^b^ before HIV diagnose	26/28^a^	93
Tested for HIV before	25/30	83
Steady partner at time of interview	13/30	43

^a^Data on education and prior STI diagnosis from 2 participants are lacking

^b^STI Sexually transmitted infections (chlamydia, gonorrhea, and syphilis) cART combination Anti Retroviral Therapy

Except for one participant, all men engaged in sexual contact in the first year after HIV diagnosis. At time of HIV diagnosis, 13 men were involved in a steady relationship, and they all reportedly disclosed their HIV status to their steady partner. Overall, the first occurrence of anal sexual contact (with or without condom use) after the HIV diagnosis varied from the day of diagnosis to over 1 year after diagnosis.

## Motives for condom use

Table 2 provides an overview of the motives and barriers identified in this study. Half of the 29 participants who had anal intercourse in the first year following HIV diagnosis reported using a condom at least once. From the interviews with these participants, we identified 10 distinct motivational aspects for condom use in three categories: motives to protect the sex partner from HIV, motives to protect oneself, and situational triggers.

**Table 2:** Motives and barriers for condom use and regular STI testing

	Condom use	Regular STI testing
Motives	*Protecting the sex partner (20)* ^a^ Preventing further transmission Learning from your own mistakes Protecting the less experienced Emotional bond *Personal benefit (8)* Protection for STI Alternative to disclosure Habitual condom use Hygienic benefits *Situational triggers (7)* Mutual decisions with sexual partners Compliance with situational norms	*Health consciousness (15)* *Protecting partner from STI (5)* *Feeling vulnerable for infections (7)*
Barriers	*Emotional reactions to HIV diagnosis (8)* Feelings of vulnerability and worthlessness Anger for being infected Relief from fear of HIV *Transition from HIV-negative to -positive (10)* Difficulties in changing sexual behaviour patterns Insisting on condom use seen as indirect disclosure No perceived need for self-protection *Interpersonal and situational factors (11)* Assumed concordant HIV status Casual sexual partner’s decision Steady partner’s wish Using drugs/alcohol in a sexual context	*Perceptions of safety (10)* Misperceptions STI transmission risk Misperceptions of STI testing procedures *Situational barriers (5)* Aversion to STI testing site Burdensome testing procedures

^a^ (x) numbers of participants addressing these topics during the interviews

### Protecting the sex partner

Four motivational aspects for condom use that were intended to protect the sex partner from HIV infection are described below.

#### Preventing further transmission

Some men reported a personal norm not to infect others as a reason for condom use.

*Yes, I made a decision: you may not infect somebody. You are playing with lives although, yes, life expectancies are good nowadays—but it is a traumatic thing you will inflict upon somebody, and it also causes social suffering. No, I think you have to carry your responsibility and use a condom. (RDB013)*

*What I noticed in the gay scene is that people act like, ‘well I’m throwing the condom away, I have HIV, so it doesn’t matter anymore, carpe diem.’ Not that I would do such a thing but I hear it around me. But this is not the world I want to enter, it offends me: so this is how far you would go for personal sexual satisfaction (all the risks they will take). It’s a kind of lovelessness and hardness what I do not understand and it makes me angry and upset. (RDB014)*

#### Learning from your own mistakes

Another motive to use a condom was based upon participants’ own experience—how they thought they had been infected with HIV. This raised their awareness of HIV transmission, and they translated this awareness into taking extra care with condoms and other preventive behaviours with sex partners.

*Condoms, yes, during fucking always. I take extra care now, so it doesn’t go wrong with friends. And so I won’t come in their mouth any more; this is also risky, I now think.*

*Now? (interviewer)*

*It [HIV infection] happened to me that way. (RDB010)*

#### Protecting the less experienced

A younger age of the sex partner sometimes evoked extra motivation for protective measures. Participants considered partners too young to fully comprehend the risk of not using a condom. The following quote came from a participant who was infected at 25–30 years of age, which made him draw the line at that age.

*That is why actually I do not want to have sex with people who are, for example, younger than 30 years. I think that when you are 30, you can weigh out the risks of unsafe sex, but before that you are a little too thoughtless. (RDB003)*

#### Emotional bond

Several men felt extra protective towards somebody they cared for.

*If I fuck somebody, and it is somebody I have some feelings for, I could not bear the thought that I perhaps did not use all the options of protection.*

*What is the difference? (interviewer)*

*Because you bond with that person. Just, you’re more concerned for his wellbeing. (RDB009)*

### Personal benefit

We identified four motivational aspects for condom use that were of personal added value.

#### Protection from STI

Men were motivated to use condoms because they feared contracting another STI and/or had the notion of being more vulnerable to STI due to the HIV diagnosis.

*Personally, I protect myself against STI and, I do not know exactly, possibly also against herpes or another type of HIV, because it seems there are 2 or 3 types of HIV, details I don’t know. But anyway, I think having one [*i.e. *HIV] is already bad enough.. (RDB023)*

*But yes, now I am afraid that when I contract an STI, that my CD4 cells..uh..well after those antibiotics I had to take, those CD4 cells were very bad. (RDB003)*

#### Alternative to disclosure

Some men used condoms as a way to avoid disclosure of their HIV status. They preferred to keep their status private and feared gossip and rejection.

*I prefer not to tell, but I take care that I have sex as safe as possible. Then I don’t have to tell, right? I mean, if I have sex as safe as possible, then it isn’t necessary to tell? (RDB012)*

*I didn’t tell him, I let him believe I always use a condom and have safe sex, well then I don’t have to tell him because I feared the confrontation and being rejected. (RDB002)*

#### Habitual condom use

Some men stated that condom use was a customary behaviour before their HIV diagnosis, and their attitude towards condom use was positive. For some of these men, the HIV diagnosis did not change this behaviour.

*From the moment of my first sexual encounter—I was like 18 years or so—using a condom was the norm. That’s imprinted. So although I am involved in a steady relationship, I…(also out of hygienic reasons or so, I think). Yes, always a condom. (RDB009)*

#### Hygienic benefits

Some men mentioned hygiene as a motive for condom use.

*Condoms often make you feel like you’re dealing with STI the whole time, and that’s not the 100% free feeling. However it is also good; I mean, it has a feeling of hygiene to use condoms, and that’s what I do like. I am somebody who loves clean and fresh. Sex with poop I don’t like at all, it feels dirty. So the advantage is, if accidently there is some poop on it, on the condom, just remove the condom and really, that’s what makes me feel clean again. Even the smell of poop makes me lose my erection. (RDA002)*

### Situational triggers

We identified two main triggers for condom use: mutual decision between the sex partners and situational cues.

#### Mutual decisions with sexual partners

Sometimes a decision to use condom was based on a discussion that took place prior to the sexual act. In the following specific case the disclosed HIV status, care status, and the fear of re-infection played a role in the subsequent decision making:

*And how did you come to that decision that condoms should still be used? (interviewer).*

*Well, I do not know exactly how it went, but there was an issue with him using medication already and I did not, so we did not want to have problems with another infection or re-infection, something like that. I had a young infection and he already for some time. So we felt it was not wise to not use a condom.*

*And you had it figured out before you had sex? (interviewer)*

*Yes, actually it was a very natural logical step. (RDB023)*

Some participants mentioned they always disclosed their HIV status before sex and then decided whether or not to have condomless sex based on the reaction to this disclosure, the discussion that sometimes followed, or the related decision taken by the sex partner.

Some men used condoms only if requested by their sex partner.

*Yes, I am honest, if someone asks me directly.*

*How did he react? (interviewer)*

*He said: then we will use a condom. I said, ‘That’s fine.’ (RDB0008)*

#### Compliance with situational norms

Some social situations dictate customary sexual protective behaviours, e.g. a sex party or sex venue where condom use is the explicit norm.

*All sex parties I know have clear rules: safe sex only. You can even be expelled if you do not follow them. (RDA014)*

## Barriers to condom use

Almost half of the participants reported condomless anal sex in the first year following HIV diagnosis. Interviews with these men indicated 10 barriers to condom use during anal intercourse in the first year, divided into three distinct groups: (1) barriers related to emotions, (2) barriers related to the transition from an HIV-negative to an HIV-positive serostatus, and finally, (3) situational barriers related to the characteristics of the sexual partnerships/contacts or place of sexual contact.

### Emotional reactions to HIV diagnosis

#### Feelings of vulnerability and worthlessness

Some men felt vulnerable after their diagnosis, experiencing a loss of self-esteem. They feared rejection if their HIV status became known and could not bear the consequences of people knowing their HIV status.

*I felt like a lame horse going into battle, a worthless being, so really I don’t think I could cope if somebody rejected me. In the beginning I couldn’t handle this at all. Actually I needed, I accepted, any kind of sex—whatever the other person wanted, with or without [protection]. (RDB0211)*

#### Anger

Some men expressed anger towards the person from whom they believed they had acquired the HIV infection. One man explicitly mentioned that this caused him to be frustrated and disappointed and to therefore engage in condomless sex.

*First I was stunned, then I grew furious. Why should I care for others if they didn’t care for me? It took me two weeks to calm down, but I am not proud of what I did in those weeks. (RDA017)*

#### Relief

Some men said that being diagnosed with HIV put an end to their sexually active life of watching out for HIV, being always careful during sexual acts. They felt they had gained freedom from the fear of contracting HIV.

*I must admit that I also felt a relief, never to be afraid anymore, as I always have been for HIV. I became less strict in condom use.” (RDB009)*

### The transition from HIV-negative to -positive

For some men, the HIV diagnosis came as a shock. They had to cope with a life-changing situation and needed time to adapt and integrate HIV into their sexual life.

#### Difficulties in changing sexual behaviour patterns

Some men were, prior to the HIV diagnosis, involved in condomless anal intercourse with a regular sex partner. Some of them experienced difficulties in changing the pattern of condomless sex out of fear they could be challenged to disclose their HIV status.

*Soon after, I didn’t know how to change. He would wonder why I would want him to use a condom; I couldn’t change that, and continued [in condomless sex]. Now, I seldom have sex anymore: only anonymous partners and only safe. Not with people I know, because they would expect me to do it unsafe. I’d rather not have to explain that I contracted it. (RDA014)*

#### Insistence on condom seen as indirect disclosure

One man remembered when, being HIV-negative, he rejected sexual contact with a man who insisted on using condoms. His opinion was that men who insist on condom use with HIV-negative men must be HIV-positive. On becoming HIV-positive himself, this opinion persisted.

*Before, when somebody else stressed condom use, -even though I told them I was checked and was negative, I thought they must have something bad, and I certainly would not go through with the sex…So stressing on condoms for me now is like telling everybody I’m HIV-positive.” (RDA 011)*

#### No perceived need for self-protection

Some men did not see any added value in using condoms. For example, they did not feel the need to protect themselves against STIs. Although aware of STIs, they felt that it would not matter to have a minor other infection besides the ‘worst of them all.’

*I’ve gotten more careless towards condoms. I think, ‘You already have HIV.’ Yes, this is the worst what could happen to me, it has become reality and I am still alive. It’s a funny feeling. You survived. Yes, you survived. (RDB0066)*

### Interpersonal and situational factors

#### Assumed concordant HIV status

Some barriers for condom use were based on the interaction between the sex partners, the HIV status of the partner, and the context of the sexual encounter. A perceived concordant HIV-positive status of the partner was often mentioned as a reason to engage in condomless sex.

*Well I did think like this, ‘I can fuck unsafe, but not with everybody.’ I do watch out with whom. Yes, if I think people don’t have it, then I am not engaging in unsafe sex just like that.*

*How do you find out? (interviewer)*

*Actually, you do not really know. (RDA019)*

*Well, he clearly wanted to have sex without a condom so he must have been HIV-positive, or else how stupid can you be? (RDB0008)*

#### Casual sexual partner’s decision

Some men said that, having disclosed their HIV-positive status, it was up to the sex partner to decide on condom use.

*It’s more up to the other. If the other says, ‘I want to use a condom,’ that’s fine with me. If the other says, ‘I want without a condom,’ I say, ‘But I am sero-positive, think twice.’ Then sometimes you hear them say, ‘Yes, I am as well.’*

*And if they don’t? (interviewer)*

*Then I think, ‘You’re old and wise enough.’ You know, these are not twenty-year-olds. They are forty or maybe fifty. You know enough. (RDB003)*

*To make it easier and more honest and to avoid problems, I tell beforehand, 9 out of 10 times, especially* via *internet. If I want to date someone, I want to know how he feels about HIV-positive men, because I am HIV-positive. (RDA014)*

#### Steady partner’s wish

In general, the wish to protect an HIV-negative steady partner was strong. However, during a long-term relationship men sometimes engaged in condomless sex. This was related to the desire of the HIV-negative partner to have condomless sex.

*He sometimes wants to have sex without a condom. Well, we have discussions. He says that when he’s top the risk is very small, but I don’t believe this…But we sometimes engage in unprotected sex.*

*How does this make you feel? (interviewer)*

*Well, mixed feelings. On one hand I think it’s his own responsibility, especially if he consciously does not use them; but on the other hand, I would feel very bad if he turns out to be infected at one time. (RDA0016)*

#### Using drugs/alcohol in a sexual context

Alcohol and drug use were not uncommon among participants, both before and after the HIV diagnosis. Some men reported the difficulty of maintaining condom use when intoxicated, but stated that having sex while intoxicated was less stressful and made them worry less about transmission risks.

*It makes it [sex] easier, yes. Also in the future on the occasion that I use drugs and there is the opportunity to fuck unsafe, then it is a lot easier. (RDA019)*

## Motives for regular STI testing

Most of the men had visited STI/HIV testing services more than once before their HIV diagnosis. All but one man reported having had an STI before the HIV diagnosis: mostly gonorrhoea, chlamydia and or syphilis. In the year after HIV diagnosis, only a few men reported regular STI testing. Most men who were recently diagnosed had not yet gone for STI testing, and those who did, went irregularly. A total of 37% (11/30) of all the men reported having had an STI after being diagnosed with HIV. We identified four motives for testing and five barriers to testing.

### Health consciousness

Most men were trying to manage their health when diagnosed with HIV. For some, one way to manage their health was to get regularly tested for STIs, trying to avoid having STI-related problems or an STI that goes unnoticed.

*It’s good to have it all under control and I go every 3 months, so it should be under control and stays within limits (RDB023).*

*You go every 6 months? (interviewer to RDB002)*

*Yes, certain things you don’t notice you have them. It’s nice that they…that it will be fixed. (RDB002)*

### Protecting partner from STI

By getting checked regularly, men tried to protect their sex partner for STI.

*I mean, if you have casual contacts, then it’s kind of a duty to go and do this, for yourself but also for others. (RDB035)*

*Well, at a certain moment we had a more open relationship, so we thought we have to do it [testing] again. Because for me as well as for my partner, it’s yes: you don’t know what others do and you hear more and more about STI. And I thought, let’s do it at least once a year standard. Just to be sure—and I have a partner and things can go wrong. It’s better to know. For me it’s like this: we have more often sex with others, and to prevent that we give each other something and ping-pong infections, it’s good to do it, but I did not have any symptoms or anything. (RDB012)*

### Feeling vulnerable for infections

Some men said that they tended to be more alert to physical complaints soon after their diagnosis, compared to the period before. Increased notions of vulnerability led to regular and sometimes more frequent STI clinic visits.

*Every 6 months, yes. Like last time: I had a bit of a sore throat and, having a cold or something, it does triggers something and I get paranoia: O God this is the end. And because I do blowjobs without a condom, I thought, ‘Oh sore throat, I got something,’ and immediately went for check-up, but it was nothing—just a common cold. (RDB013)*

## Barriers to regular STI testing

Some men had justified reasons for not testing for STI, considering they had no sexual contact. However, for the majority of sexually active men, barriers to regular testing were divided into perceptions regarding risks and situational factors.

### Perceptions of safety

While we did not conduct a systematic inventory of correct or incorrect knowledge of STIs during the interviews, in some of the cases such specific knowledge or lack of it was indicated by participants as a barrier for proper STI testing.

#### Misperceptions about STI transmission risk

Some men felt protected against STIs when using condoms and therefore did not go for regular testing.

*I did want to come [for testing] regularly, especially when I had more sexual contacts, because I heard you can have an STI without symptoms. But you know, I thought, well I am doing it safe. (RDA002).*

#### Misperceptions about testing procedures

Men receiving HIV care sometimes had the false belief that they were checked for all STIs as part of their routine health controls at the HIV care centres. Consequently they felt safe and free of STI. However, presently, HIV clinics do not screen for bacterial STIs like gonorrhea and chlamydia.

*Yes, I get checked regularly, at the HIV clinic. In the beginning I went often and at this moment 2 or 3 times a year. About 6 litres of blood they draw every time, and the whole story gets checked, so for lues, other STI and HIV blood values, but also sugar etc. etc. (RDB0211)*

### Situational barriers

#### Aversion to STI testing site

Some men hesitated to return to the location where they received the HIV diagnosis and to face the health care personnel.

*Now that I have HIV, I did not go back to this place again. Not because they did a bad job here. I don’t know, it is so complex. Looking back, I think they did a very good job, human, kind, supportive. But I don’t want to see her anymore. I did not want to get the test result from anybody else but her, but I get nervous thinking about her. Why should I come back? For STI testing? I am doubtful, I don’t want to go but I am not trusting X either. I also think because I have HIV, all the rest is less important. (RDA 008)*

*Yes, it is a confrontation; I did not have the urge or necessity to come here again. No, and it is, to be honest, a bad place. Yes, it has a negative association. (RDA002)*

#### Burdensome testing procedures

Waiting time, as well as the time it takes to complete STI testing procedures, were mentioned as barriers.

*That’s the main issue, I think: it takes such a long time, you will have to spend the whole morning or evening. (RDA006)*

*It stops you from regular testing? (Interviewer to RDB023.)*

*Yes, even if you have a privileged appointment, even then it is, yes it’s just a long wait. And that’s awkward, all those people. (RDB023)*

## Discussion

The purpose of this study was to gain insight into motives and barriers to condom use and regular STI testing among MSM soon after HIV diagnosis.

Motives for condom use were based on the wish to protect the sex partner, perceived personal benefits, and situational triggers. Motives relating to the wish to protect the sex partner included, for example, a feeling of responsibility for less-experienced sex partners and, in general, for preventing further transmission of HIV. Motives related to personal benefits included self-protection from STI [25] and perceived hygienic benefits, but also condom use as an alternative to disclosure of HIV status. Situational triggers included mutual decisions between sex partners and compliance with situational norms. Motives for regular STI testing were a need for control over ones health, a feeling of increased vulnerability to STIs, and a desire to protect sexual partners.

The motives we found towards compliance with desirable sexual health behaviours are the key to motivational communication with our target group, as they represent readily accepted reasons to engage in these behaviours. Our findings still need to be corroborated by quantitative studies, but our list of reasons why men choose to use condoms or test for STI soon after diagnosis can form the backbone of any intervention rationale to persuade these men to adopt these behaviours. However, the barriers we found towards these behaviours represent the greatest challenge for developing such persuasive communication.

We found that many emotions such as anger, relief, and vulnerability were expressed by our participants post-diagnosis. These emotions, each in its own unique way, have negatively influenced the intrinsic motivation towards condom use. Following IMB principles, interventions should anticipate these emotions and help men to process them and to understand that, while usually temporary, they can influence behaviour in negative ways.

As for the choices we have made in the recruitment process, our assumption that recruiting men who were diagnosed longer than 1 year before the interview will help yield rapport that reflects deeper understanding of barriers and motives has been corroborated by our participants. To quote one men with diagnosis time 1> :

*Well actually I did not have any sex at all, in the beginning. Also because I didn’t feel sexy at all. And that’s very important; you don’t feel desirable because you do not feel mentally good at all. The effect of giving it a place, the effect it has on your live, that it is do-able, you notice that it [HIV infection] is not completely all comprehensive, as it has been. That you still continue you’re your work, keep the same contacts, the same house. (RDB002).*

Not only emotions played an important role in the decision-making of our participants. Our men realized that although their sexual reference context had changed enormously, their sexual patterns and habits had not. Habits which were not harmful to others when one was HIV-negative had suddenly become harmful post-diagnosis. Preventive interventions should invest in helping men to change prior patterns of sexual risk behaviour according to their new reality. According to the IMB model, intervention efforts will need to enhance negotiation and communication skills to cope with changing social-sexual challenges such as manoeuvring in groups, dealing with community pressures, and managing interpersonal relationships [26–28].

The environmental and the situational facets of sexual encounters also play a role in the ability of men to protect others and themselves. In our sample, as in other populations, alcohol and drugs were linked in a special way to HIV diagnosis [29]. Substance use is a way of escaping the implications of the diagnosis, and at the same time it forms an important barrier to protective behaviours. This should be acknowledged in interventions. More awareness of risk and skills to improve self-control during drug use are also important components in any future intervention that aims to support men after diagnosis.

As for STIs, men had certain assumptions about whether or not they are at risk. For some, low perceptions of STI risk were the reason for not testing. For some, these perceptions could be valid, but for many they are unwarranted. For example, men who engaged in frequent sex with different partners, but always used a condom, often assumed they did not need to test for STI, although infections can be acquired even with condom use. Furthermore, some participants incorrectly thought that they were tested for all STIs during visits to the HIV clinic, and therefore had a false sense of security. Those perceptions could be counteracted by addressing and explaining the testing procedures for STI during visits at the HIV specialist.

It is important for preventive interventions post-diagnosis to sort out and correct problematic risk perceptions regarding STIs among the newly diagnosed. That can be done by direct communication by health professionals who convey clear messages and/or provide motivational counseling when indicated.

The facilities for STI testing also form a barrier for some men. Some found the testing procedures burdensome (e.g. inconvenient opening hours) or expressed a strong negative association with a facility because it was the place they were diagnosed with HIV. On the organizational level, following principles of the Sorensen model of incorporating social context in health behaviour interventions, facilities can do better to accommodate these patients [24, 30]. In addition, facilities can organize alternative options, such as home-based STI testing, that do not necessitate coming to the clinics.

Regarding study limitations, homosexual men of Dutch origin dominated the study sample. We did not include bisexual men or more than a few men of non-Dutch origin. Bisexual men and men originating from other countries may face problems not typical of our sample, and future research should explore the additional motives and barriers to condom use and regular testing that may exist in these groups [31, 32].

The field of HIV is rapidly changing. Men are increasingly diagnosed earlier and also treated earlier, and consequently the impact of transmissibility will change. However, the fact remains that HIV diagnoses are not always immediately followed by treatment, [33–35] and once treatment is initiated, weeks or even months can elapse before the drugs can eliminate viral load [36]. The finding that condomless sexual contact occurred from the same day of diagnose onwards, emphasizes that even with the prospect of effective treatment as prevention there are and probably will always be incidents of condomless anal sex post diagnosis that pose actual threat of HIV transmission.

That interim period is crucial for preventing further transmission of HIV as well as co-infection with other STIs (especially syphilis, hepatitis B and C, and Lymphogranuloma venereum) that might facilitate HIV-transmission or complicate the HIV treatment and prognosis. In addition the recent developments in preventive biomedical interventions could also influence sexual behavior post diagnosis with HIV negative partners using pre exposure prophylaxis (Prep). Men recently diagnosed not yet on treatment might indeed feel more comfortable having sexual contact with other men using PrEP. This prospect of ‘PrEP-sorting’, should be a topic that merits research in the future. Furthermore the perspective of being virally suppressed and hence non infectious once cART is initiated could also play an alleviating role in the perceptions of men recently diagnosed. Especially as these perceptions are now glowingly being recognized and accepted by HIV negative men. This topic also merits further research attention.

## Conclusions

MSM recently diagnosed with HIV need to deal with their new health condition and its implications. It is in this period, directly following the HIV diagnosis, that new perspectives on sexuality and new sexual behavioural patterns may be formed. Timely support could strengthen the ability of recently diagnosed men to make better-informed decisions regarding their sexual health. Considering the great diversity of motives and barriers to condom use and STI testing, professionals involved in their care are challenged to accommodate men individually with the information, motivation, and skills that match their personal needs. We believe that an intervention that will be adaptable and carefully tailored to the personal situation of each newly infected individual can be a promising tool of support. Such interventions can be provided through personal counselling or through tailored computer-based interventions online or offline.

## Abbreviations

ACS: Amsterdam Cohort Studies
cART: Combination anti-retroviral therapy
CINIMA: Center for Infection and Immunology Amsterdam
HIV: Human Immuno-deficiency Virus
i.e.: id est, it is, for example
IMB: Information Motivation Behaviour skills model
IQR: Interquartile range
MSM: Men who have sex with men
PHSA: Public Health Service of Amsterdam
STI: Sexually transmitted infection
UAI: Unprotected Anal Intercourse
